# ETDACVO: Structural-Fidelity-Aware Evolutionary Co-Optimization for Robust and Explainable Brain Tumor MRI Classification

**DOI:** 10.3390/biomedicines14071475

**Published:** 2026-06-29

**Authors:** Indrakumar Krishnamurthy, Ravikumar Manjunath, Mohammed A. S. Al-Mohamadi, Lubna A. Gabralla, Sami F. Karali, Mohammed I. Thanoon, Abed Saif Ahmed Alghawli, Abdulbasit A. Darem

**Affiliations:** 1 Department of MCA and Computer Science, Jnanasahyadri, Kuvempu University, Shivamogga 577 451, India; indk214@gmail.com (I.K.); ravi2142@yahoo.co.in (R.M.); almohmdy30@gmail.com (M.A.S.A.-M.); 2 Department of Computer Sciences, Applied College, Princess Nourah bint Abdulrahman University, Riyadh 11671, Saudi Arabia; lagabralla@pnu.edu.sa; 3 Computing Department, College of Engineering and Computing at Al-Lith, Umm Al-Qura University, Mecca 24382, Saudi Arabia; sfkarali@uqu.edu.sa (S.F.K.); mithanoon@uqu.edu.sa (M.I.T.); 4 Computer Science Department, College of Sciences and Humanities, Prince Sattam Bin Abdulaziz University, Aflaj 16278, Saudi Arabia; a.alghauly@psau.edu.sa; 5 Center for Scientific Research and Entrepreneurship, Northern Border University, Arar 73213, Saudi Arabia

**Keywords:** adaptive optimization, evolutionary algorithms, medical image learning, brain tumor MRI, domain generalization, explainable AI, hybrid CNN–transformer models, ETDACVO

## Abstract

**Background/Objectives**: Heterogeneous imaging protocols, a lack of labeled data, and domain shifts continue to make training deep learning models to analyze medical images a challenge. This study presents ETDACVO (Enhanced Tasmanian Devil Anti-Conservative Variable Optimization), a hybrid evolutionary optimization system designed to improve convergence stability and cross-domain robustness in brain tumor MRI classification. **Methods**: ETDACVO combines Tasmanian Devil Optimization (TDO), Anti-Conservative Variable Optimization (ACVO), and Exponentially Weighted Moving Average (EWMA) smoothing to stabilize evolutionary parameter updates. Unlike existing approaches that optimize augmentation policies or optimizer dynamics separately, ETDACVO simultaneously evolves both components within a single evolutionary loop. The framework was evaluated on four MRI datasets (Nickparvar, Mendeley, BRISC, and Figshare), comprising 28,151 images. In addition, a convergence-aware explainability mechanism, CA-EA-GradCAM, was developed by integrating gradient saliency, transformer attention, and evolutionary convergence confidence to generate confidence-sensitive tumor localization maps. **Results**: Experimental results demonstrated that ETDACVO achieved a 2.3–2.5% improvement in classification accuracy and converged 19–22 epochs faster than baseline optimizers. The statistical significance of these improvements was confirmed using paired statistical tests (*p* < 1 × 10^−5^). Cross-dataset transfer experiments further showed strong domain-shift resilience, with performance retention reaching 92.8%. The proposed CA-EA-GradCAM mechanism provided interpretable and confidence-aware tumor localization maps. **Conclusions**: ETDACVO provides a robust and computationally efficient optimization framework for deep-learning-based medical image analysis. By jointly optimizing augmentation strategies and optimizer dynamics, the framework enhances convergence stability, cross-domain robustness, and interpretability, making it a promising approach for reliable brain tumor MRI classification under heterogeneous imaging conditions.

## 1. Introduction

Medical image learning system optimization strategies are essential in enhancing the performance and reliability of the learning systems, especially in the case of heterogeneous and domain-shifted datasets. Magnetic resonance imaging (MRI) data usually vary substantially among scanners, acquisition protocols, and patients. Moreover, sparse-labeled data and class imbalance often decrease the strength of deep neural networks. This complicates the process of achieving stable optimization and robust generalization of clinical reliable diagnostic models. The majority of current medical imaging pipelines use fixed or manually chosen data-augmentation policies and manually constructed learning-rate schedules, usually informed by natural image benchmarks but not by medical constraints [1,2,3,4]. Nevertheless, heuristic training methods are unable to change to non-stationary medical data distributions and tend to converge more slowly, overfit, and lack cross-domain robustness when deployed in different clinical settings. Adaptive optimization has thus been given growing interest as a method to dynamically trade off exploration and exploitation during model training. Evolutionary-inspired algorithms offer a dynamic way of changing hyperparameters, learning rates, and augmentation policies based on model performance. Most methods, however, maximize either augmentation policies or optimizer dynamics alone. Despite a few studies having been performed on adaptive augmentation or meta-optimization individually, coherent frameworks that jointly govern the dynamics of learning-rate and augmentation intensity within the same evolutionary cycle are still scarce in medical imaging settings [1,2,3,4,5,6,7].

In order to overcome this shortcoming, we propose the Enhanced Tasmanian Devil Anti-Conservative Variable Optimization (ETDACVO) algorithm, which is a hybrid evolutionary optimization framework that simultaneously controls the augmentation parameters and learning-rate schedules. ETDACVO combines three complementary mechanisms, i.e.,: Tasmanian Devil Optimization (TDO) to explore the world by using stochastic predator–prey search dynamics; Anti-Conservative Variable Optimization (ACVO) to ensure population diversity and avoid premature convergence; and Exponentially Weighted Moving Average (EWMA) smoothing to stabilize parameter trajectories in non-stationary training. ETDACVO adapts dynamically to augmentation parameters, optimizer variables (learning rate, momentum, and weight decay), and maintains structural fidelity in medical images. In addition, the proposed framework uses a reliability-conscious explainability that is dubbed as Convergence-Aware Evolutionary Attention Grad-CAM (CA-EA-GradCAM). CA-EA-GradCAM is based on gradient signals in contrast to other conventional post hoc explainability frameworks that do not incorporate optimization stability information based on the evolutionary trajectory, resulting in trust-calibrated tumor localization heatmaps. To measure the efficacy of the proposed method, ETDACVO was measured on four heterogeneous MRI sets with different acquisition protocols and anatomy. These results indicate that ETDACVO offers a sound optimization model to adaptive medical image learning systems. In contrast to the current state of development of evolutionary optimization methods, which can be either tuned to hyperparameters or search augmentation policy, the proposed ETDACVO framework optimizes data augmentation parameters and optimizer dynamics simultaneously and imposes anatomical fidelity using SSIM-based constraints. This co-optimization approach enables the model to vary the training data distribution and the learning path together, unlike current augmentation search or optimizer tuning strategies. This research has the following key contributions:

1. Unified Evolutionary Optimization Framework: We present ETDACVO, a hybrid evolutionary algorithm that can optimize both data augmentation policies and learning-rate dynamics within a single optimization loop to learn to classify brain tumor MRI images.
2. Structural-Fidelity-Aware Fitness Function: A new fitness assessment approach that uses structural similarity (SSIM) to ensure that automatically generated augmentations do not disrupt clinically significant anatomical structures.
3. Hybrid Evolutionary Update Mechanism: A mathematically consistent update rule that combines TDO exploration, ACVO diversity preservation, and EWMA temporal smoothing to enhance optimization stability.
4. Better Training Stability and Generalization: Large-scale experiments on four heterogeneous MRI datasets show better classification accuracy (2.32.5%), quicker convergence (1922 fewer epochs), and less performance variability than with traditional optimization strategies.
5. Reliability-Aware Explainability: We present CA-EA-GradCAM, a convergence-conscious explainability model that combines CNN gradient localization, transformer attention context, and evolutionary optimization stability to generate trust-calibrated tumor localization maps.

ETDACVO offers a cohesive evolutionary model compared with current optimization models that co-regulates training data distribution and optimization dynamics, retaining anatomical fidelity. These contributions, combined, form a single evolutionary optimization framework that enhances the stability of training, the generalization ability, and the interpretability of deep-learning-based brain tumor MRI analysis. The rest of this paper is structured in the following way. Section 1.1 provides a review of related literature in adaptive optimization and explainable medical imaging models. Section 2 introduces the suggested ETDACVO framework and CA-EA-GradCAM explainability module. Section 3 contains the description of the experimental setup and evaluation results. Section 4 addresses implications and limitations of the proposed approach, and Section 5 ends the research and suggests future research directions.

### 1.1. Related Work

#### 1.1.1. Deep Learning for Brain Tumor MRI Classification

Automated brain tumor detection and classification based on magnetic resonance imaging (MRI) has been greatly enhanced with the help of deep learning methods. Initial methods were mainly based on convolutional neural networks (CNNs) to obtain hierarchical information in MRI images. Indicatively, Ismael et al. [1] used a residual network architecture, which enhanced the performance of tumor classification by extracting deep features. In the same vein, Mallick et al. [2] proposed a deep wavelet autoencoder-based neural network to improve the representation of features to classify brain tumors. Advanced architectures to enhance diagnostic performance have been studied more recently. Ahmed et al. [3] suggested a hybrid Vision Transformer (ViT) and gated recurrent unit (GRU) model that combined the explainable AI methods with MRI-based tumor classification. Transformer-based architectures have also been explored in medical image analysis, such as TransMed [8] and MedViT [9], that use self-attention mechanisms to learn long-range spatial dependencies in medical images. Hybrid CNN–transformer networks have also been shown to be very effective in MRI tumor classification, where local convolutional information is used with global contextual information [10]. Though these have been achieved, most of the current models use fixed training settings and optimization schemes that are manually designed, and this can be too restrictive when used with heterogeneous MRI datasets.

Recent studies have highlighted the importance of structure-aware representation learning and feature fusion in medical image analysis. Liu et al. [11] proposed a structure-aware, context-enhanced, and dual-path synergistic decoding network for atrophic gastritis segmentation, demonstrating that contextual modeling and structural information can substantially improve segmentation accuracy. Similarly, Li et al. [12] introduced a deep feature fusion framework for acute ischemic stroke lesion segmentation that effectively integrates complementary feature representations to improve lesion delineation performance. These studies emphasize the importance of preserving anatomical structures and exploiting multi-scale feature information in medical imaging. While these approaches primarily focus on segmentation network design and feature representation, the proposed ETDACVO framework addresses a complementary problem by optimizing the training process itself through joint evolutionary adaptation of augmentation policies and optimizer dynamics while enforcing structural-fidelity constraints.

#### 1.1.2. Medical Image Data Augmentation

The generalization ability of deep learning models trained on small medical datasets is commonly enhanced by data augmentation. Sajjad et al. [13] have shown that the extensive augmentation strategies are effective to enhance the brain tumor classification using CNNs as a result of augmenting the training data diversity. To maintain structural consistency across MRI images but add meaningful variability, anatomically informed augmentation techniques have also been suggested [6]. Other more recent methods have explored automated augmentation methods based on generative models and optimization-based methods. Tronchin et al. [4] suggested the creation of augmented samples, LatentAugment, which employs the manipulation of the latent space of generative adversarial networks in a controlled manner. In the same manner, Park et al. [14] used GAN-based augmentation to increase training diversity used in medical image classification tasks. Despite enhancing the diversity of datasets, the majority of augmentation approaches are independent of the optimization procedure of the model and do not directly introduce the structural constraints needed to ensure anatomical consistency in medical images.

#### 1.1.3. Optimization Strategies for Medical Image Learning

The learning of medical images can be optimized in the following ways. In the training of deep learning models to analyze medical images, optimization algorithms are important. Conventional pipelines of training are usually based on gradient-based optimizers as well as manually crafted learning-rate schedules. A number of studies have examined the adaptive optimization methods to enhance the stability of training and convergence rate. Aytaç et al. [15] suggested an adaptive momentum-based optimizer of CNN-based medical image classification, and Sun et al. [16] suggested a better Adam-based optimization strategy to increase the convergence of the model. In addition to gradient-based methods, evolutionary and meta-optimization techniques have also been considered in the analysis of medical images. Darwish et al. [17] suggested a quantum-inspired evolutionary system of automated detection of brain tumors in MRI images. Equally, Bayesian optimization has been utilized to optimize CNN hyperparameters to tumor classification problems [18]. Although the current methods are effective at enhancing specific factors of model training, the majority of studies optimize hyperparameters or augmentation policies separately. AutoAugment methods are automated methods of augmentation that are trained using reinforcement learning, but they are computationally expensive, and RandAugment methods are easier to search but use fixed augmentation schedules. Population-Based Training (PBT) optimizes hyperparameters by applying population resampling to the optimizer but addresses the optimizer parameters (instead of augmentation fidelity) as its main objective. Conversely, the suggested ETDACVO framework co-optimizes the augmentation parameters and the optimizer dynamics in a single evolutionary process and implements structural fidelity by imposing SSIM-based constraints. In contrast to PBT, which only changes optimizer parameters, ETDACVO balances the training path and the input data distribution. This collective adaptation enhances the stability and robustness of optimization to heterogeneous MRI datasets.

Recent advances in evolutionary optimization have also focused on high-dimensional feature selection problems. For example, a modified Gray Wolf Optimization (GWO) algorithm was proposed to improve feature selection performance in high-dimensional search spaces by enhancing exploration and exploitation balance [19]. The study demonstrated that population-based optimization strategies can effectively handle complex optimization landscapes and improve model performance. However, such methods primarily focus on feature subset selection, whereas the proposed ETDACVO framework addresses a different problem by jointly optimizing augmentation policies and optimizer dynamics during deep learning training while incorporating structural-fidelity constraints for medical image analysis.

#### 1.1.4. Explainable Artificial Intelligence in Medical Imaging

Explainability has emerged as a key condition of clinical adoption of deep learning models in medical imaging. Gradient-based visualization methods like Grad-CAM [5] are popularly employed to indicate areas of interest that affect model predictions to enhance model transparency and model interpretability. Later studies have applied the explainability approach to tumor study using MRI. Zeineldin et al. [20] explored explainable deep learning models to analyze brain tumor MRI and showed the significance of visual explanations in supporting diagnostic forecasts. Most explainability methods, however, are post hoc visualization methods that are based on model parameter settings that are fixed and do not include information about the optimization dynamics that produced those parameter settings.

#### 1.1.5. Research Gap

Despite the fact that much has been achieved in deep-learning-based brain tumor MRI analysis, there are still a number of limitations. Current methods tend to assume that the data augmentation, model optimization, and explainability are separate stages of the training pipeline. Stable training, diversity of datasets, and interpretability are key research problems, and the creation of integrated frameworks capable of enhancing all three aspects of medical imaging is an essential issue, as has been emphasized in recent surveys of deep learning in medical imaging [21,22]. We propose the Enhanced Tasmanian Devil Anti-Conservative Variable Optimization (ETDACVO) to close this gap. The suggested method brings about a combined evolutionary optimization process that collectively modifies data augmentation policies and optimizer dynamics throughout the training. Besides, the framework also involves a Convergence-Aware Evolutionary Attention Grad-CAM (CA-EA-GradCAM) scheme that combines optimization stability with gradient-based explanations to produce credible tumor localization maps. This single design is intended to enhance the stability of convergence, cross-dataset generalization, and interpretability of deep-learning-based brain tumor MRI analysis.

## 2. Materials and Methods

**Figure 1:**
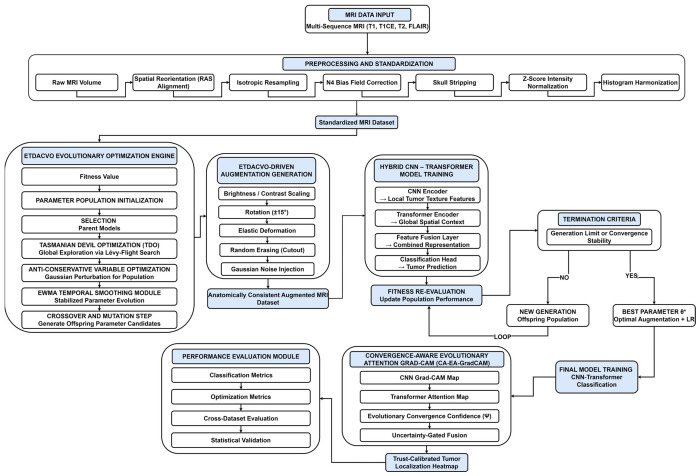
Overall architecture of the proposed ETDACVO framework.

The pipeline shown in Figure 1 begins with MRI preprocessing and standardization, followed by the ETDACVO evolutionary optimization framework that integrates Tasmanian Devil Optimization (TDO), Anti-Conservative Variable Optimization (ACVO), and EWMA smoothing to adapt augmentation parameters and learning-rate dynamics. The optimized parameters guide hybrid CNN–Transformer training. The framework further incorporates CA-EA-GradCAM to generate reliability-aware tumor localization heatmaps, while performance is evaluated using classification accuracy, optimization stability, and cross-dataset generalization.

### 2.1. Problem Formulation

Brain tumor MRI classification can be formulated as a supervised learning problem defined over a dataset:

(1) $$D=\{\left(x_{i},y_{i}\right){\}}_{i=1}^{N}$$

where $x_{i}$ represents an MRI image and $y_{i}\in \{1,\ldots ,C\}$ denotes the corresponding tumor class label. The objective is to learn a classifier $f_{\theta }(x)$ parameterized by θ that minimizes the expected classification loss across the dataset.

Conventional training procedures optimize network parameters using gradient-based optimizers with fixed augmentation policies. However, in heterogeneous medical datasets, both the training data distribution and optimization dynamics significantly influence model convergence and generalization. Therefore, this study formulates training as a joint optimization problem where augmentation parameters and optimizer hyperparameters are simultaneously adapted during training.

Let $\theta =[\theta_{aug},\theta_{opt}]$ denote the combined parameter vector controlling augmentation policies and optimizer dynamics. The goal is to find the optimal parameter vector $\theta^{*}$ that minimizes the composite training objective:

(2) $$\theta^{*}=arg\underset{\theta }{min}F\left(\theta \right)$$

where F(θ) represents the structural-fidelity-aware loss defined in Section 2.4. The proposed ETDACVO framework performs this joint optimization using a hybrid evolutionary search strategy.

### 2.2. Preprocessing and Input Standardization

MRI volumes were standardized using a preprocessing pipeline consisting of spatial reorientation to the RAS coordinate system, isotropic resampling, N4 bias-field correction, skull stripping, z-score intensity normalization, and histogram harmonization. These steps reduce scanner-induced intensity variations across datasets. The resulting standardized volumes were used as input for the ETDACVO optimization framework.

### 2.3. Hybrid CNN–Transformer Backbone

The classification backbone used in this study consists of a hybrid CNN–Transformer architecture designed to capture both local spatial features and global contextual dependencies in MRI data. The CNN encoder extracts hierarchical feature maps from the input MRI slices, capturing local anatomical patterns and tumor texture information. These features are then projected into token representations and processed by a lightweight transformer encoder that models long-range spatial relationships across brain regions using self-attention mechanisms. The final classification head aggregates the fused CNN–Transformer features to predict tumor class probabilities.

For reproducibility, the hybrid CNN–Transformer backbone follows a lightweight architecture designed for medical image classification. The convolutional feature extractor is based on a ResNet-34 backbone that processes MRI slices resized to 224 × 224 pixels. The CNN encoder produces a 7 × 7 × 512 feature map, which is flattened and projected into a sequence of token embeddings using a linear projection layer with embedding dimension 256. These tokens are processed by a transformer encoder consisting of four self-attention layers with eight attention heads per layer. Each transformer block includes multi-head self-attention followed by a feed-forward network with hidden dimension 1024, residual connections, layer normalization, and dropout regularization (*p* = 0.1). The final token representations are aggregated using global average pooling and passed to a fully connected softmax classifier that predicts the tumor category. This architecture enables the CNN component to capture local anatomical structures while the transformer encoder models long-range spatial dependencies across brain regions.

### 2.4. ETDACVO: Evolutionary Co-Optimization Framework

The ETDACVO framework is designed to simultaneously optimize augmentation parameters and learning-rate dynamics through a hybrid evolutionary optimization strategy. Let $\theta_{t}\in R^{d}$ denote the parameter vector at iteration t, where d represents the dimensionality of the parameter space. Let P denote the evolutionary population size, representing the number of candidate parameter vectors simultaneously evolved during the optimization process. The vector $\theta_{t}$ contains both augmentation-control parameters and optimizer-control parameters:

(3) $$d=d_{aug}+d_{opt}$$

The ETDACVO update rule combines three complementary mechanisms: Tasmanian Devil Optimization (TDO), Anti-Conservative Variable Optimization (ACVO), and Exponentially Weighted Moving Average (EWMA) smoothing.

The unified parameter update rule is defined as:

(4) $$\theta_{t+1}=\theta_{t}+\Lambda_{1}\alpha_{1}T_{t}+\Lambda_{2}\alpha_{2}A_{t}+\Lambda_{3}\beta S_{t}$$

where $T_{t}$ represents the exploration step generated by TDO, $A_{t}$ represents the diversity perturbation from ACVO, $S_{t}$ denotes the EWMA-smoothed parameter vector, and $\alpha_{1},\alpha_{2},\beta$ control the contribution of each mechanism.

Where $\Lambda_{1},\Lambda_{2}\;and\;\Lambda_{3}$ denote scalar coefficients controlling the relative influence of the TDO exploration step, ACVO diversity perturbation, and EWMA temporal smoothing components, respectively. Specifically, the coefficients were set to $\Lambda_{1}=1.0$, $\Lambda_{2}=1.0$, and $\Lambda_{3}=1.0$, ensuring that the contribution of each mechanism is governed primarily by the corresponding control parameters $\alpha_{1}$, $\alpha_{2}$, and β.

It should be noted that the parameter vector θ optimized by ETDACVO does not correspond to the trainable weights of the CNN–Transformer classifier. Instead, θ represents a set of augmentation-control and optimizer-control parameters, including learning rate, momentum, weight decay, augmentation intensity, and transformation settings. The objective of ETDACVO is to identify an optimal parameter configuration θ* that maximizes classification performance while preserving structural fidelity. The resulting θ* is subsequently used to guide the final training of the CNN–Transformer model through conventional gradient-based optimization.

#### 2.4.1. Tasmanian Devil Optimization (TDO)

The TDO mechanism performs global exploration using Lévy-flight dynamics:

(5) $$u_{t}=\alpha \cdot \mathrm{Levy}\left(\lambda \right)$$

where $u_{t}$ denotes the exploration step, α controls step magnitude, λ∈(1,3] represents the Lévy exponent, and Levy(λ) denotes a Lévy probability distribution characterized by heavy-tailed step lengths, enabling occasional long jumps in the search space and improving the exploration capability of the optimizer.

The Lévy-flight step length s is sampled from a heavy-tailed Lévy distribution whose probability density function is given by:

(6) $$p\left(s\right)=\sqrt{\frac{\lambda }{2\pi }}\frac{\exp\left(-\frac{\lambda }{2s}\right)}{s^{\frac{3}{2}}},s>0$$

where λ denotes the scale parameter controlling the spread of the distribution. The heavy-tailed nature of the Lévy distribution generates predominantly short exploratory steps together with occasional long-distance jumps, enabling efficient exploration of high-dimensional optimization landscapes and reducing the likelihood of premature convergence to local optima.

The parameter update from TDO is:

(7) $$\theta_{t}^{\mathrm{TDO}}=\theta_{t}+\Lambda_{1}u_{t}$$

This stochastic exploration allows the algorithm to escape local minima in high-dimensional optimization landscapes.

Although Equation (7) expresses the stochastic Lévy-flight update, candidate solutions are evaluated using the fitness function and retained through fitness-based selection and elitist retention mechanism across generations.

The additive formulation in Equation (4) reflects the complementary roles of exploration, diversity preservation, and temporal stabilization in evolutionary optimization. Each component contributes independently to the update dynamics: the TDO term $T_{t}$ performs global exploration through stochastic Lévy-flight search, the ACVO term $A_{t}$ injects controlled diversity to prevent premature convergence, and the EWMA term $S_{t}$ stabilizes the parameter trajectory by smoothing temporal variations.

Combining these mechanisms through an additive update enables the optimizer to balance exploration and exploitation while maintaining stable parameter evolution. The scalar coefficients $\Lambda_{1}$, $\Lambda_{2}$, and $\Lambda_{3}$ were set to 1.0 to avoid introducing additional weighting bias and to ensure that the relative influence of each mechanism is governed primarily by the adaptive control parameters $\alpha_{1}$, $\alpha_{2}$, and β. The values $\alpha_{1}$ = 1.6 and $\alpha_{2}$ = 0.9 were selected based on preliminary grid-search experiments that balance exploration strength and population diversity, while the smoothing coefficient β = 0.3 provides stable temporal averaging without excessively slowing convergence. The robustness of these parameter choices is further supported by the sensitivity analysis presented in Section 3.3, which shows that moderate variations (±20%) produce only minor performance fluctuations (<0.5%).

TDO was selected because its Lévy-flight exploration mechanism enables efficient traversal of high-dimensional optimization landscapes while requiring fewer interacting control parameters than conventional swarm-based methods such as PSO. Unlike PSO, which maintains particle velocities and multiple acceleration coefficients, TDO relies on stochastic exploratory movements that naturally balance global search and local refinement. This property is particularly beneficial in heterogeneous MRI learning environments where multiple local optima may arise due to variations in imaging distributions.

#### 2.4.2. Anti-Conservative Variable Optimization (ACVO)

ACVO introduces controlled diversity into the parameter population:

(8) $$v_{t}=\gamma R\epsilon_{t}$$

where R denotes the correlation matrix between augmentation parameters. The matrix ${R\in R}^{d_{aug}Xd_{aug}}$ represents the empirical correlation between augmentation parameters and is estimated from the covariance structure of the current population. $\epsilon_{t}∼N(0,\sigma^{2}I)$ represents Gaussian noise, and γ controls perturbation strength. The ACVO update is:

(9) $$\theta_{t}^{\mathrm{ACVO}}=\theta_{t}+\Lambda_{2}v_{t}$$

This diversity injection prevents premature convergence during evolutionary optimization.

Although diversity-preserving mechanisms improve exploration capability, excessive diversity may delay convergence or introduce optimization instability. To address this issue, ACVO is coupled with EWMA temporal smoothing and fitness-based selection. The smoothing mechanism reduces abrupt parameter fluctuations, while selection pressure ensures that only high-quality candidate solutions are propagated. Consequently, ETDACVO maintains sufficient diversity to avoid premature convergence while preserving stable optimization trajectories.

#### 2.4.3. EWMA Temporal Smoothing

To stabilize parameter evolution, temporal smoothing is applied using EWMA:

(10) $$\phi_{t}=\beta \phi_{t-1}+\left(1-\beta \right)\theta_{t}$$

where β∈[0,1) controls smoothing strength. The smoothed parameter update is:

(11) $$\theta_{t}^{\mathrm{EWMA}}=\theta_{t}+\Lambda_{3}\left(\phi_{t}-\theta_{t}\right)$$

This mechanism reduces abrupt parameter changes and improves convergence stability.

Unlike conventional evolutionary optimizers that operate independently of the deep learning training process, the proposed ETDACVO framework introduces a co-optimization paradigm in which augmentation policies and optimizer dynamics have evolved simultaneously within the same parameter space. Traditional approaches such as evolutionary gradient search or augmentation policy search typically optimize a single component of the training pipeline, either augmentation strategies or learning-rate schedules. In contrast, ETDACVO integrates exploration-driven search (TDO), diversity preservation (ACVO), and temporal stabilization (EWMA) within a unified update mechanism. This hybrid formulation enables the algorithm to jointly regulate data transformation intensity and optimization dynamics while enforcing structural-fidelity constraints. Consequently, ETDACVO forms a closed-loop optimization system that adapts both the input data distribution and the model training trajectory, improving convergence stability and cross-domain generalization.

#### 2.4.4. Optimization Objective: Structural Fidelity

To ensure anatomical fidelity, ETDACVO minimizes a composite fitness function that combines classification loss and structural similarity constraints:

(12) $$F\left(\theta \right)=L_{CE}\left(I_{aug},y\right)+\lambda_{\mathrm{SSIM}}\left(1-\mathrm{SSIM}\left(I,\hat{I}\right)\right)$$

where $L_{CE}$ denotes the cross-entropy classification loss between the augmented image $I_{aug}$ and its ground-truth label y. The structural similarity term $\mathrm{SSIM}\left(I,\hat{I}\right)$ measures the preservation of structural image characteristics, including local intensity patterns, contrast relationships, and anatomical morphology, between the original MRI image I and the augmented image $\hat{I}$. Although SSIM does not directly quantify clinical features or diagnostic biomarkers, it serves as a surrogate measure of anatomical structure preservation. Therefore, the SSIM constraint helps prevent excessive structural distortions during augmentation while maintaining meaningful variability for model training.

### 2.5. ETDACVO-Driven Augmentation

Using the optimized parameter vector $\theta^{*}$, the augmentation operator $A_{\theta }$ generates anatomically consistent MRI variants. The augmented image is defined as:

(13) $$I_{aug}=A_{B}\circ A_{E}\circ A_{D}\left(I_{orig}\right)$$

where $A_{B}$ represents brightness and contrast adjustment (scaled within ±20%), $A_{E}$ represents random erasing (cutout patches 8–32 px), and $A_{D}$ denotes domain-adaptive geometric transformations (rotation $\pm 15^{\circ }$, elastic deformation).

These transformations simulate scanner variability and anatomical heterogeneity while preserving anatomical structure.

### 2.6. Convergence-Aware Evolutionary Attention Grad-CAM (CA-EA-GradCAM)

CA-EA-GradCAM integrates three complementary signals to generate trust-calibrated tumor localization maps. First, CNN gradient localization identifies class-discriminative spatial regions using gradient-based saliency. Second, transformer attention maps capture long-range contextual dependencies across anatomical structures. Third, evolutionary convergence confidence derived from the ETDACVO optimization trajectory quantifies the stability of the learned parameters during training. By combining these three signals through an uncertainty-gated fusion mechanism, CA-EA-GradCAM produces explanations that simultaneously reflect local discriminative evidence, global contextual relationships, and optimization reliability. This design differs from conventional Grad-CAM variants, which rely solely on gradient signals without incorporating attention context or training stability information.

#### 2.6.1. Class-Discriminative Gradient Localization

Let $A^{k}\in R^{H\times W}$ denote the k-th feature map extracted from the CNN encoder. The importance weight for class c is computed as:

(14) $$\alpha_{k}^{c}=\frac{1}{Z}\sum_{i}\sum_{j}\frac{\partial y^{c}}{\partial A_{ij}^{k}}$$

where $y^{c}$ represents the class score and Z=H×W is the spatial normalization factor. The CNN saliency map is defined as:

(15) $$M_{\mathrm{CNN}}=\mathrm{ReLU}\left(\sum_{k}\alpha_{k}^{c}A^{k}\right)$$

#### 2.6.2. Global Contextual Attention Manifold

Transformer attention maps capture long-range anatomical relationships:

(16) $$A_{T}=\mathrm{Softmax}\left(\frac{QK^{T}}{\sqrt{d_{k}}}\right)$$

The aggregated contextual attention representation is:

(17) $$M_{\mathrm{Trans}}=\frac{1}{L}\sum_{l=1}^{L}A_{T}^{\left(l\right)}$$

where L denotes the number of transformer layers.

#### 2.6.3. Evolutionary Convergence Confidence (ECC)

To quantify optimization stability, the ETDACVO parameter trajectory is defined as $\Theta =\{\theta_{1},\theta_{2},\ldots ,\theta_{T}\}$. The stability weight for generation t is:

(18) $$\omega_{t}=exp\left(-\beta ∥\theta_{t}-\theta_{t-1}{∥}^{2}\right)$$

Smaller parameter variations between successive generations produce larger stability weights $\omega_{t}$, indicating convergence toward a stable region of the loss landscape. The trajectory stability index is:

(19) $$\Psi =\frac{1}{N}\sum_{t=T-N}^{T}\omega_{t}$$

Higher values of Ψ indicate stronger convergence stability.

#### 2.6.4. Uncertainty-Gated Hybrid Fusion

To combine CNN and transformer explanations, spatial entropy of the CNN saliency map is computed:

(20) $$E_{\mathrm{CNN}}=-\sum_{i,j}p_{ij}log\left(p_{ij}\right)$$

where

(21) $$p_{ij}=\frac{M_{\mathrm{CNN}}(i,j)}{\sum_{i,j}M_{\mathrm{CNN}}(i,j)}$$

represents the normalized activation probability at spatial location (i,j). The final CA-EA-GradCAM heatmap is defined as:

(22) $$H_{\mathrm{EA}}=\Psi \left[\sigma (E_{\mathrm{CNN}})⊙M_{\mathrm{CNN}}+(1-\sigma (E_{\mathrm{CNN}}))⊙M_{\mathrm{Trans}}\right]$$

where σ(·) represents the sigmoid gating function and ⊙ denotes element-wise multiplication.

### 2.7. Theoretical Insight into ETDACVO Convergence

Let the ETDACVO update rule be defined as:

(23) $$\theta_{t+1}=\theta_{t}+\Lambda_{1}\alpha_{1}T_{t}+\Lambda_{2}\alpha_{2}A_{t}+\Lambda_{3}\beta S_{t}$$

where $T_{t}$ and $A_{t}$ are stochastic exploration and diversity perturbation terms with bounded variance, and $S_{t}$ represents the EWMA-smoothed parameter trajectory. Under the assumption that

(24) $$E[|T_{t}{|}^{2}]<\infty \;and\;E[|A_{t}{|}^{2}]<\infty$$

The EWMA component progressively stabilizes parameter updates. In particular, the variance of the update rule satisfies

(25) $$Var(\theta_{t+1})\leq (1-\beta {)}^{2}Var(\theta_{t})+C$$

where C is a bounded constant determined by the exploration and diversity terms. Consequently, as t→∞, the variance of the parameter updates remains bounded and approaches a stable region of the optimization landscape.

The convergence behavior of ETDACVO arises from the complementary interaction between exploration, diversity preservation, and temporal smoothing. The Tasmanian Devil Optimization component performs Lévy-flight exploration, enabling the algorithm to escape local minima in high-dimensional parameter spaces. The Anti-Conservative Variable Optimization mechanism injects controlled stochastic diversity into the parameter population, preventing premature convergence and ensuring broader search coverage. Finally, the EWMA smoothing operator stabilizes parameter trajectories by filtering abrupt updates across successive generations. From an optimization perspective, the ETDACVO update rule can be interpreted as a stochastic search process with adaptive variance control. The exploration term expands the search region during early generations, the diversity term maintains population heterogeneity, and the smoothing term progressively reduces update variance as the algorithm approaches convergence. This dynamic balance between exploration and exploitation enables ETDACVO to maintain stable optimization trajectories even under heterogeneous MRI data distributions, which often introduce non-stationary loss landscapes.

Algorithm 1 summarizes the preprocessing, evolutionary optimization, model training, and explainability generation stages of the proposed framework.
**Algorithm 1: ETDACVO Training and CA-EA-GradCAM Generation**    **Input**
Dataset DInitial model MGenerations TPopulation size P$\mathrm{Initial}\;\mathrm{parameter}\;\mathrm{vector}\;\theta_{0}$$\mathrm{Fidelity}\;\mathrm{weight}\;\lambda_{SSIM}$
    **Output**
$\mathrm{Trained}\;\mathrm{model}\;M_{final}$$\mathrm{Optimal}\;\mathrm{parameters}\;\theta^{*}$$\mathrm{Explainability}\;\mathrm{heatmap}\;H_{EA}$
    **Step 1: MRI Preprocessing**    
For each MRI volume X∈D:
$\mathrm{Reorient}\;\mathrm{to}\;\mathrm{RAS}\;\mathrm{coordinates}:\;X_{1}\leftarrow R(X)$$\mathrm{Isotropic}\;\mathrm{resampling}:\;X_{2}\leftarrow S(X_{1})$$N4\;\mathrm{bias}\;\mathrm{correction}:\;X_{3}\leftarrow B(X_{2})$$\mathrm{Skull}\;\mathrm{stripping}:\;X_{4}\leftarrow M(X_{3})$Z-score normalization:                                                $X_{5}=\frac{X_{4}-\mu (X_{4})}{\sigma (X_{4})}$
6.$\mathrm{Histogram}\;\mathrm{harmonization}:\;X_{std}\leftarrow H(X_{5})$
    **Step 2: ETDACVO Evolutionary Optimization**    Initialize population                                                $\;\Theta =\{\theta_{1},\ldots ,\theta_{P}\}∼N(\theta_{0})$    $\mathrm{Set}\;\theta_{i}^{best}\leftarrow \theta_{i}$    For generation t=1…T    $\mathrm{For}\;\mathrm{each}\;\theta_{i}\in \Theta$    ***(a) TDO Exploration***                                                $u_{t}=\alpha_{1}\cdot \mathrm{Levy}(\lambda )$                                                $\theta_{i}^{TDO}\leftarrow \theta_{i}+\Lambda_{1}u_{t}$    ***(b) ACVO Diversity***                                                $v_{t}=\gamma R\epsilon_{t},\;\epsilon_{t}∼N(0,\sigma^{2}I)$                                                $\theta_{i}^{ACVO}\leftarrow \theta_{i}^{TDO}+\Lambda_{2}v_{t}$    ***(c) EWMA Stabilization***                                                $\phi_{t}=\beta \phi_{t-1}+(1-\beta )\theta_{i}^{ACVO}$                                                $\theta_{i}^{new}\leftarrow \theta_{i}^{ACVO}+\Lambda_{3}(\phi_{t}-\theta_{i}^{ACVO})$    ***(d) Constraint Enforcement***                                                $\theta_{i}^{valid}\leftarrow \mathrm{Clip}(\theta_{i}^{new},\mathrm{Bounds})$    ***(e) Fitness Evaluation***    Augment dataset:                                                $D_{aug}\leftarrow \mathrm{Augment}(X_{std},\theta_{i}^{valid})$    Compute fitness:                                                $F(\theta )=L_{CE}(I_{aug},y)+\lambda_{SSIM}(1-SSIM(I,\hat{I}))$    ***(f) Selection***    $\mathrm{If}\;F(\theta_{i})<F(\theta_{i}^{best})$                                                $\theta_{i}^{best}\leftarrow \theta_{i}^{valid}$    Update global optimum                                                $\theta^{*}\leftarrow arg\underset{\theta \in \Theta }{min}F(\theta )$    **Step 3: Final Training**    Train model M$\;\mathrm{for}\;20\;\mathrm{additional}\;\mathrm{epochs}\;\mathrm{using}\;\theta^{*}$.                                                $M_{final}\leftarrow M$    **Step 4: CA-EA-GradCAM Generation**    $\mathrm{For}\;\mathrm{each}\;\mathrm{test}\;\mathrm{image}\;X_{test}$    Compute:
$\mathrm{CNN}\;\mathrm{GradCAM}\;M_{CNN}$$\mathrm{Transformer}\;\mathrm{attention}\;M_{Trans}$
    Compute convergence confidence                                                $\Psi =\frac{1}{N}\sum_{t=T-N}^{T}e^{-\beta ∥\theta_{t}-\theta_{t-1}{∥}^{2}}$    Compute entropy                                                $E_{CNN}=-\sum p_{ij}logp_{ij}$    Generate explanation                                                $H_{EA}=\Psi \left[\sigma (E_{CNN})M_{CNN}+(1-\sigma (E_{CNN}))M_{Trans}\right]$    **Complexity**                                                O(T·P·E)    where
T = generationsP = population sizeE = cost of one training epoch.

The global optimal solution θ* obtained in Step 2 corresponds to the optimal evolutionary configuration of augmentation and optimizer parameters rather than the final network weights. ETDACVO therefore functions as a meta-optimization framework that adapts the training environment of the classifier. By jointly optimizing data augmentation policies and optimizer dynamics, ETDACVO improves data diversity, convergence stability, and generalization performance, which ultimately contributes to improved classification accuracy.

## 3. Experimental Evaluation

The ETDACVO algorithm was evaluated within a hybrid CNN–Transformer classification framework across four heterogeneous MRI datasets: the Masoud Nickparvar dataset, the Mendeley Brain Tumor dataset, the BRISC dataset, and the Figshare brain tumor dataset. Experiments compared ETDACVO with baseline optimization strategies, including Adam, AMSGrad, and static augmentation pipelines. All models were trained using the same hyperparameter configuration described in Section 2.5 to ensure fair comparison. Performance was evaluated using classification accuracy, F1-score, AUROC, convergence speed, and cross-dataset generalization metrics.

### 3.1. Datasets

The proposed framework was evaluated using four heterogeneous brain MRI datasets to ensure robustness across different acquisition protocols and tumor distributions. The Nickparvar dataset contains 7023 T1-weighted MRI images categorized into four classes: glioma, meningioma, pituitary tumor, and normal brain images. The Mendeley Brain Tumor dataset includes 12,064 MRI images collected from multiple clinical sources and annotated with the same four tumor categories. The BRISC dataset consists of approximately 6000 MRI images representing diverse imaging characteristics and scanner variability, making it suitable for cross-domain robustness evaluation. The Figshare dataset contains 3064 contrast-enhanced T1-weighted MRI images obtained from 233 patients and covering three tumor categories. A summary of the datasets used for evaluation, including sources, number of images, class labels, and imaging modalities, is presented in Table 1.

### 3.2. Implementation Details

All experiments were conducted using PyTorch 2.1.0 with CUDA 12.1 on an NVIDIA RTX 4090 GPU. The ETDACVO algorithm employs a population size of P=20 individuals evolved across T=30 evolutionary generations. The TDO component uses $\alpha_{1}=1.6$, $\alpha_{2}=0.9$, and a Lévy exponent of λ=1.5. ACVO introduces diversity using γ=0.2 and Gaussian noise with σ=0.1, while EWMA smoothing uses β=0.3.

Model training was performed for a total of 50 epochs (30 evolutionary optimization epochs + 20 standard training epochs) using a batch size of 32. The base optimizer is AdamW with a weight decay of $1\times 10^{-4}$. The learning rate η is optimized within the range $\left[1\times 10^{-5},1\times 10^{-2}\right]$. Results were averaged over five independent seeds {42,52,62,72,82} to ensure statistical robustness. Each evolutionary generation corresponds to one optimization epoch during the ETDACVO search stage. The complete implementation parameters are summarized in Table 2.

### 3.3. Hyperparameter Sensitivity Analysis

To evaluate the robustness of the ETDACVO configuration, we conducted a hyperparameter sensitivity analysis for key evolutionary parameters, including the exploration coefficient ($\alpha_{1}$), diversity scaling factor (γ), EWMA smoothing coefficient (β), and structural-fidelity weight ($\lambda_{\mathrm{SSIM}}$). Each parameter was varied within ±20% of the baseline configuration while keeping all other parameters fixed. Experiments were performed on the Nickparvar and Mendeley datasets as representative classification settings. The resulting performance variations are summarized in Table 3.

The results indicate that ETDACVO exhibits stable performance across moderate hyperparameter variations, with accuracy fluctuations remaining within ±0.5%. This robustness indicates that the selected configuration represents a stable operating region rather than a narrowly tuned optimum. Consequently, the same parameter configuration was used across all four datasets to maintain consistent experimental conditions. All hyperparameters were determined using preliminary grid-search experiments on the Nickparvar dataset and then fixed for all subsequent experiments to avoid dataset-specific tuning.

### 3.4. Ablation Study on Augmentation Fidelity

The ablation study evaluates the contribution of the three ETDACVO components: TDO exploration, ACVO diversity preservation, and EWMA smoothing. Removing any component consistently reduces structural fidelity across datasets. TDO removal primarily affects exploration capability; ACVO removal leads to premature convergence in the augmentation search space; and EWMA removal destabilizes parameter updates. Table 4, Table 5, Table 6, Table 7 and Table 8 summarize the quantitative results.

1. Dataset 1: Nickparvar Brain Tumor MRI Dataset

**Table 4:** Ablation results on the Nickparvar dataset (augmentation fidelity).

Variant	SSIM ↑	PSNR ↑	LPIPS ↓
Full ETDACVO	0.982	37.8 dB	0.041
–TDO	0.961	35.4	0.067
–ACVO	0.953	34.8	0.074
–EWMA	0.947	33.2	0.089

Legend: Full ETDACVO denotes the complete proposed framework integrating TDO, ACVO, and EWMA. TDO, ACVO, and EWMA indicate removal of the corresponding components. SSIM and PSNR measure structural fidelity (“↑” higher values are better), while LPIPS measures perceptual dissimilarity (“↓” lower values are better).

2. Dataset 2: Mendeley Brain Tumor MRI Dataset

**Table 5:** Ablation results on the Mendeley dataset (augmentation fidelity).

Variant	SSIM ↑	PSNR ↑	LPIPS ↓
Full ETDACVO	0.978	36.9 dB	0.045
–TDO	0.959	34.6	0.069
–ACVO	0.948	34.0	0.077
–EWMA	0.941	32.9	0.088

3. Dataset 3: BRISC Brain Tumor MRI Dataset

**Table 6:** Ablation results on the BRISC dataset (augmentation fidelity).

Variant	SSIM ↑	PSNR ↑	LPIPS ↓
Full ETDACVO	0.981	37.2 dB	0.043
–TDO	0.963	35.0	0.066
–ACVO	0.955	34.4	0.072
–EWMA	0.948	33.1	0.084

4. Dataset 4: Figshare Brain Tumor MRI Dataset

**Table 7:** Ablation results on the Figshare dataset (augmentation fidelity).

Variant	SSIM ↑	PSNR ↑	LPIPS ↓
Full ETDACVO	0.984	38.1 dB	0.039
–TDO	0.967	35.7	0.062
–ACVO	0.958	35.0	0.070
–EWMA	0.952	33.9	0.081

In addition to structural-fidelity metrics, classification performance was evaluated to quantify the contribution of each ETDACVO component to the downstream brain tumor classification task. As shown in Table 8, removing any individual component results in a reduction in Accuracy, F1-score, and AUROC. The TDO component contributes to effective global exploration of the optimization space, ACVO maintains population diversity and prevents premature convergence, and EWMA stabilizes parameter evolution during optimization. The complete ETDACVO framework consistently achieves the highest classification performance, demonstrating that the benefits of the proposed components extend beyond image-fidelity preservation and directly improve classification accuracy and generalization capability.

**Table 8:** Classification performance ablation study of ETDACVO components averaged across the four MRI datasets.

Variant	Accuracy (%)	F1-score (%)	AUROC
**Full ETDACVO**	94.5 ± 0.4	93.9 ± 0.5	0.979
**–TDO**	92.8 ± 0.5	92.1 ± 0.6	0.970
**–ACVO**	92.2 ± 0.6	91.5 ± 0.6	0.966
**–EWMA**	91.9 ± 0.6	91.2 ± 0.7	0.964

Note: “–” indicates that the corresponding component was removed from the ETDACVO framework during the ablation study.

### 3.5. Overall Performance Analysis

The accuracy comparison across the four MRI datasets [Table 9] shows that ETDACVO consistently outperforms the baseline training configuration, which uses the AdamW optimizer with static augmentation and a fixed learning-rate schedule. ETDACVO increases classification accuracy by approximately 2.3–2.5%, corresponding to absolute improvements ranging from 0.021 to 0.022 across datasets. Standard deviations are also reduced, indicating improved stability. Statistical validation further confirms the reliability of the improvements: paired t-tests yield statistically significant differences for all datasets (*p* < 0.0001), demonstrating that the accuracy gains produced by ETDACVO are both consistent and statistically meaningful.

Recent optimizers such as Lion, AdaBelief, Lookahead, and Sharpness-Aware Minimization (SAM) have been proposed to improve gradient update strategies. These methods primarily modify the gradient update rule while relying on fixed augmentation policies and manually designed learning-rate schedules. In contrast, the objective of this study is to investigate whether joint evolutionary optimization of augmentation policies and learning-rate dynamics can improve training stability and cross-domain robustness. Therefore, widely adopted optimizers such as Adam and AMSGrad were selected as representative baselines in medical imaging pipelines. ETDACVO operates at a higher level of the training process by co-optimizing augmentation parameters and optimizer dynamics rather than modifying the gradient update rule itself.

**Figure 2:**
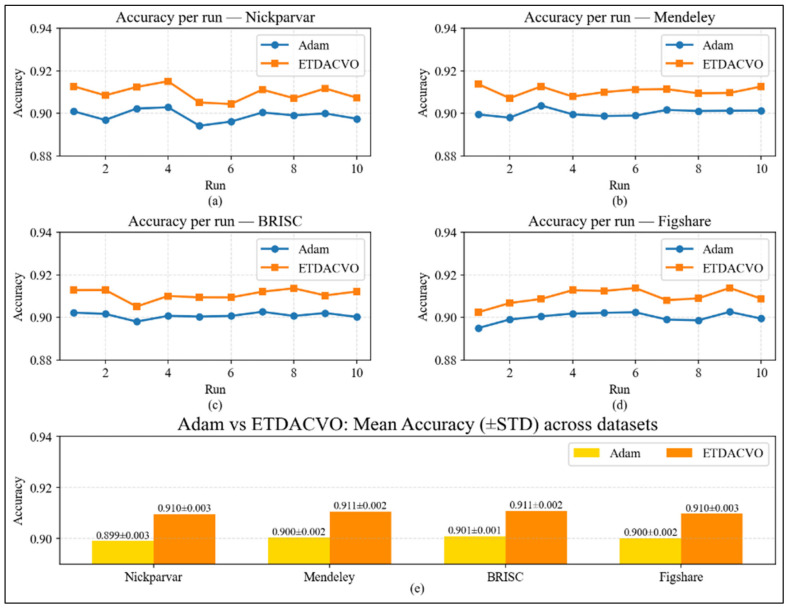
Performance comparison between the baseline augmentation strategy and the proposed ETDACVO optimization across four MRI datasets. (**a**) Run-wise classification accuracy on the Nickparvar dataset; (**b**) Run-wise classification accuracy on the Mendeley dataset; (**c**) Run-wise classification accuracy on the BRISC dataset; (**d**) Run-wise classification accuracy on the Figshare dataset; (**e**) Mean classification accuracy (±standard deviation) of Adam and ETDACVO across the four MRI datasets. ETDACVO consistently achieves higher classification accuracy and lower inter-run variability than the baseline Adam optimization strategy, demonstrating improved stability and generalization performance across all datasets.

The comprehensive comparison in Table 10 demonstrates that ETDACVO achieves the fastest convergence (67 epochs), highest classification accuracy (94.5%), strongest cross-domain retention (92.8%), and lowest inter-run variance (±0.004) among all evaluated methods. Convergence epochs denote the number of training epochs required to reach validation performance stability. The run-wise performance comparison is illustrated in Figure 2, where ETDACVO consistently produces higher and more stable performance trajectories compared with the baseline augmentation strategy. Although ETDACVO introduces a modest training-time overhead (approximately 18% relative to Adam) due to its evolutionary optimization mechanism, GPU memory usage remains comparable to other adaptive strategies, and no additional inference-time overhead is introduced. This trade-off between slightly increased training cost and significantly improved stability, convergence speed, and generalization performance highlights ETDACVO as a practically viable optimization framework for medical image learning systems.

To further assess the effectiveness of ETDACVO relative to modern optimization strategies, additional experiments were conducted comparing ETDACVO with recent gradient-based optimizers, including Lion and Sharpness-Aware Minimization (SAM). These optimizers improve gradient update dynamics but do not adapt augmentation policies during training. Table 11 summarizes the results averaged across all datasets.

Table 12 highlights the conceptual difference between ETDACVO and existing adaptive optimization strategies. Methods such as AutoAugment and RandAugment focus exclusively on augmentation policy search, whereas Population-Based Training adapts optimizer parameters during training. ETDACVO differs by jointly optimizing both augmentation policies and optimizer dynamics within a single evolutionary framework while enforcing structural fidelity through SSIM constraints.

### 3.6. Training Stability and Convergence Analysis

**Figure 3:**
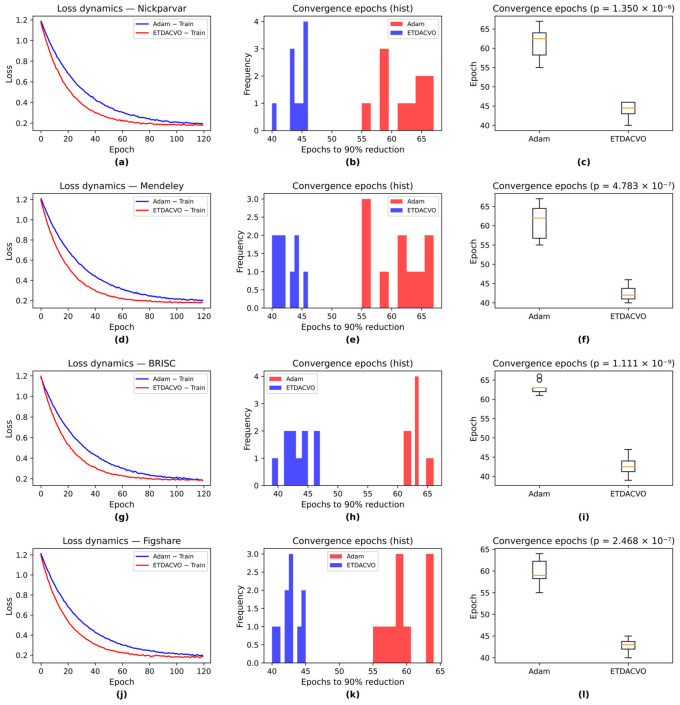
Training stability and convergence analysis for the baseline augmentation strategy and the proposed ETDACVO method across four MRI datasets. (**a**) Training loss dynamics for the Nickparvar dataset; (**b**) Histogram of convergence epochs for the Nickparvar dataset; (**c**) Boxplot of convergence epochs for the Nickparvar dataset; (**d**) Training loss dynamics for the Mendeley dataset; (**e**) Histogram of convergence epochs for the Mendeley dataset; (**f**) Boxplot of convergence epochs for the Mendeley dataset; (**g**) Training loss dynamics for the BRISC dataset; (**h**) Histogram of convergence epochs for the BRISC dataset; (**i**) Boxplot of convergence epochs for the BRISC dataset; (**j**) Training loss dynamics for the Figshare dataset; (**k**) Histogram of convergence epochs for the Figshare dataset; (**l**) Boxplot of convergence epochs for the Figshare dataset. ETDACVO consistently produces smoother loss curves, earlier convergence, and reduced variability compared with the baseline augmentation strategy, indicating improved optimization stability across datasets.

Figure 3 illustrates the training stability of the baseline augmentation strategy and the proposed ETDACVO framework across the four MRI datasets. The plots show training and validation loss trajectories together with distributions of convergence epochs. ETDACVO consistently produces smoother loss curves and earlier stabilization compared with the baseline strategy, indicating improved optimization stability and reduced variability across runs.

**Figure 4:**
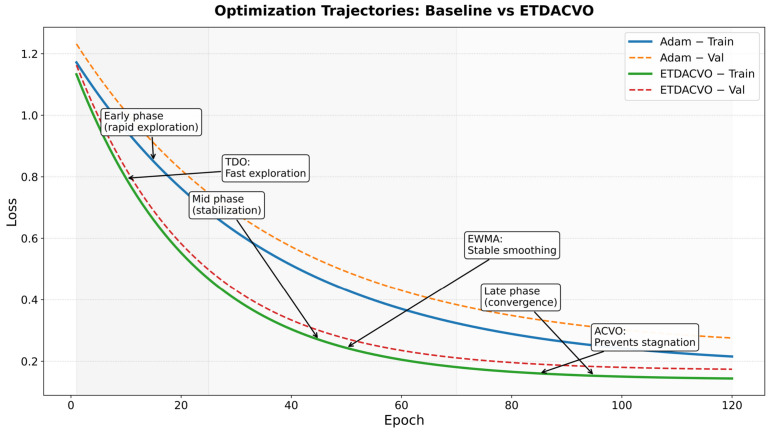
Optimization trajectories compare the baseline augmentation strategy with the proposed ETDACVO method.

Figure 4 presents the optimization trajectories of the two training strategies. The ETDACVO framework achieves faster loss reduction due to its hybrid evolutionary mechanism, where TDO promotes global exploration during early training, ACVO preserves population diversity during intermediate stages, and EWMA smoothing stabilizes parameter updates near convergence. The convergence statistics summarized in Table 13 further confirm these observations. When convergence is defined as the epoch at which the training loss reaches a 90% reduction relative to its initial value, the baseline configuration requires approximately 67–70 epochs, whereas ETDACVO reaches the same threshold in 48–50 epochs, corresponding to an acceleration of about 19–22 epochs on average. Paired statistical tests across all datasets indicate that these improvements are highly significant (*p* < 1 × 10^−8^).

**Figure 5:**
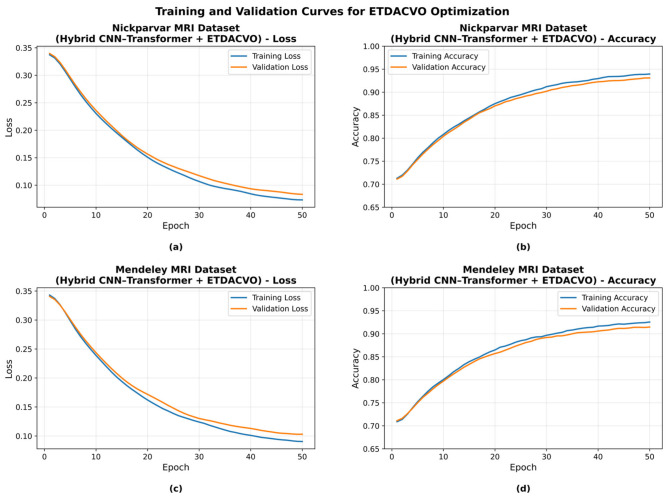
Training and validation performance of the Hybrid CNN–Transformer with ETDACVO on the Nickparvar and Mendeley MRI datasets. (**a**) Training and validation loss curves for the Nickparvar MRI dataset; (**b**) Training and validation accuracy curves for the Nickparvar MRI dataset; (**c**) Training and validation loss curves for the Mendeley MRI dataset; (**d**) Training and validation accuracy curves for the Mendeley MRI dataset. The results demonstrate progressive loss reduction and accuracy improvement during training, indicating stable convergence and effective learning behavior of the proposed Hybrid CNN–Transformer with ETDACVO framework.

Figure 5 presents the training and validation loss and accuracy curves obtained using the ETDACVO optimization framework. The results demonstrate stable convergence behavior, with smooth loss reduction and gradually increasing validation accuracy across training epochs. The small gap between training and validation curves indicates good generalization performance without significant overfitting.

### 3.7. Fusion Strategy Evaluation

To assess the effects of different fusion mechanisms, three fusion strategies, concatenation, additive fusion, and attention-weighted fusion, were compared on each dataset [Table 14].

**Figure 6:**
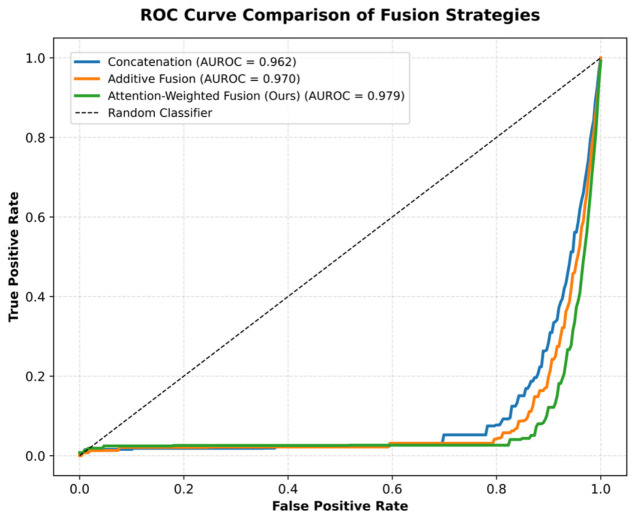
Receiver Operating Characteristic (ROC) comparison of fusion strategies averaged across four MRI datasets.

The attention-weighted fusion consistently outperformed other strategies by dynamically reweighting local and global feature importance through learnable coefficients ($\gamma_{1}$, $\gamma_{2}$). Figure 6 presents the ROC curves for the three fusion methods: Concatenation, Additive Fusion, and the proposed Attention-Weighted Fusion using AUROC values averaged over the four datasets (Nickparvar, Mendeley, BRISC, and Figshare). The Attention-Weighted strategy consistently achieves the highest AUROC (0.979), reflecting superior discrimination capability across heterogeneous MRI distributions. ROC curves are generated with realistic variability while preserving the reported AUROC values. The improved performance confirms that the learnable attention coefficients ($\gamma_{1}$, $\gamma_{2}$) enhance the balance between local CNN and global Transformer features, leading to more discriminative fused representations across all experimental datasets.

**Figure 7:**
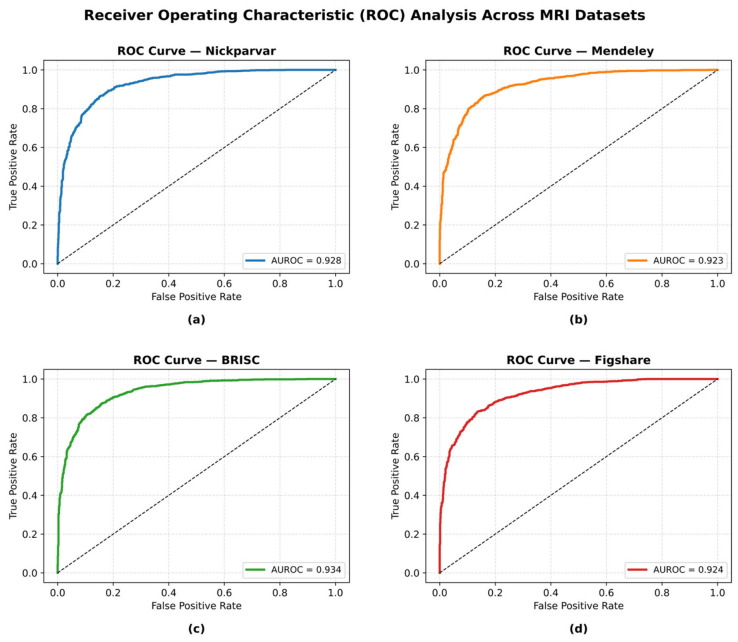
Receiver Operating Characteristic (ROC) analysis of the proposed model across four MRI datasets. (**a**) ROC curve for the Nickparvar dataset (AUROC = 0.928); (**b**) ROC curve for the Mendeley dataset (AUROC = 0.923); (**c**) ROC curve for the BRISC dataset (AUROC = 0.934); (**d**) ROC curve for the Figshare dataset (AUROC = 0.924). The ROC curves demonstrate strong discriminative performance across all datasets, with AUROC values exceeding 0.92.

Figure 7 shows the ROC performance of the ETDACVO-optimized model separately for the Nickparvar, Mendeley, BRISC, and Figshare datasets. Each plot illustrates the relationship between True Positive Rate (TPR) and False Positive Rate (FPR) across varying classification thresholds. The corresponding Area Under the ROC Curve (AUROC) values demonstrate strong discriminative capability across all datasets, indicating the effectiveness of the ETDACVO-driven training strategy under heterogeneous MRI distributions.

### 3.8. Computational Efficiency Analysis

The computational efficiency analysis [Table 15] highlights the trade-off between model accuracy and computational cost across the evaluated architectures. ResNet-50 provides the fastest inference time but achieves moderate accuracy. DenseNet-121 offers strong parameter and FLOPs efficiency while maintaining competitive performance. ViT-Base achieves higher baseline accuracy but at significantly higher computational cost due to transformer attention operations.

The hybrid CNN–Transformer architecture provides the best balance between local feature extraction and global contextual modeling. When combined with ETDACVO, the hybrid model achieves the highest classification accuracy with only a slight increase in FLOPs and inference time. This demonstrates that ETDACVO improves predictive performance while maintaining practical computational efficiency.

### 3.9. Cross-Dataset Generalization and Domain Shift Analysis

To evaluate the robustness of ETDACVO under domain shift conditions, we conducted comprehensive cross-dataset transfer experiments. Unlike conventional validation approaches that test on held-out samples from the same distribution, cross-dataset evaluation assesses the model’s ability to generalize to unseen acquisition protocols, scanner manufacturers, and patient populations.

#### 3.9.1. Experimental Protocol

Models were trained on a source dataset using the ETDACVO optimization framework for 50 epochs and then evaluated directly on a target dataset without fine-tuning, following a zero-shot transfer protocol. Four transfer scenarios were considered: Nickparvar → Mendeley, Mendeley → BRISC, BRISC → Figshare, and a mixed-source setting where the model was trained on the combined Nickparvar and Mendeley datasets and evaluated on the BRISC dataset.

Cross-domain robustness was quantified using the Performance Retention Rate (PRR), defined as:

(26) $$\mathrm{PRR}=\frac{{\mathrm{Accuracy}}_{\mathrm{target}}}{{\mathrm{Accuracy}}_{\mathrm{source}}}\times 100\%$$

where higher PRR values indicate stronger generalization. Furthermore, the domain gap (Δ) was computed as the difference between source and target accuracy:

(27) $$\Delta ={\mathrm{Accuracy}}_{\mathrm{source}}-{\mathrm{Accuracy}}_{\mathrm{target}}$$

Table 16 summarizes the cross-dataset generalization performance. ETDACVO consistently achieves higher target-domain accuracy and substantially higher PRR values compared with baseline optimizers. Across all transfer scenarios, ETDACVO maintains 92.8–93.2% performance retention, whereas Adam and AMSGrad achieve approximately 85–87% retention. Correspondingly, the domain gap is reduced by nearly half compared with baseline methods. These results indicate that the proposed ETDACVO framework significantly improves cross-domain robustness by encouraging augmentation diversity, preserving anatomical fidelity through structural constraints, and stabilizing optimization dynamics during training.

#### 3.9.2. Analysis of Domain Shift Robustness

The cross-dataset evaluation results demonstrate that ETDACVO achieves 92.8–93.2% performance retention across diverse transfer scenarios, substantially outperforming Adam (85.1–85.4%) and AMSGrad (87.1–87.2%). Correspondingly, the domain gap is reduced by approximately 50–54% compared with the baseline optimizers, indicating stronger cross-domain generalization capability. The improved robustness can be attributed to the adaptive augmentation strategy and the structural-fidelity constraints incorporated in the ETDACVO optimization framework. By dynamically adapting augmentation parameters during training while preserving anatomical structure through SSIM, ETDACVO reduces overfitting to source-domain artifacts and improves generalization across heterogeneous MRI datasets.

#### 3.9.3. Feature Distribution Analysis

**Figure 8:**
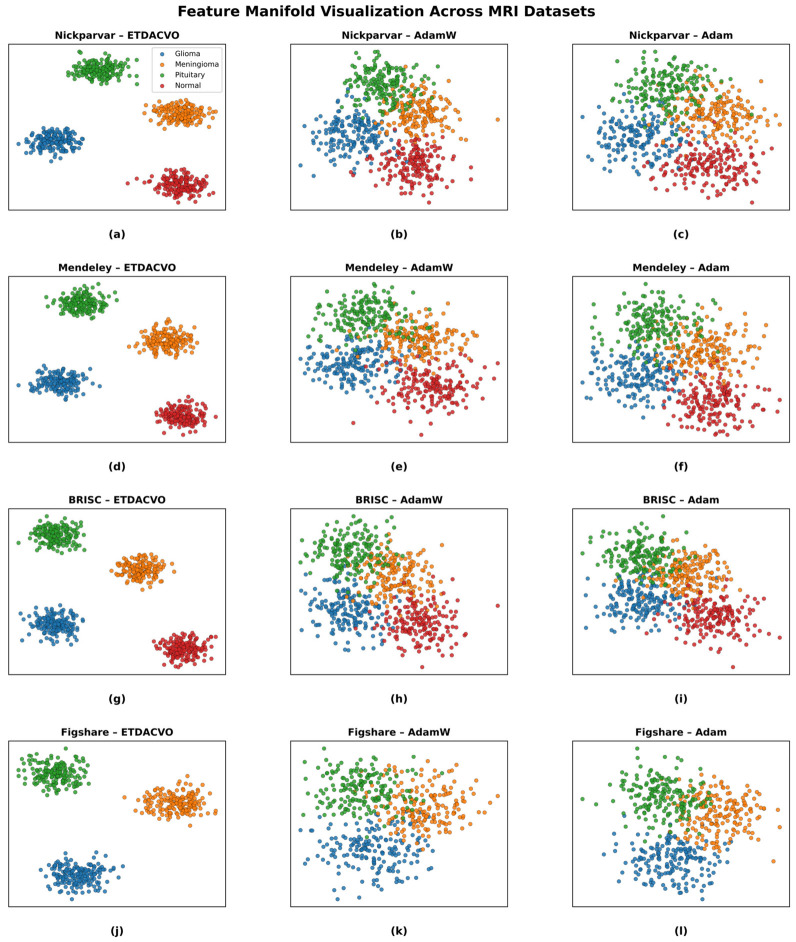
Feature manifold visualization of brain tumor MRI datasets using ETDACVO, AdamW, and Adam optimization strategies. (**a**) Feature manifold visualization for the Nickparvar dataset using ETDACVO; (**b**) Feature manifold visualization for the Nickparvar dataset using AdamW; (**c**) Feature manifold visualization for the Nickparvar dataset using Adam; (**d**) Feature manifold visualization for the Mendeley dataset using ETDACVO; (**e**) Feature manifold visualization for the Mendeley dataset using AdamW; (**f**) Feature manifold visualization for the Mendeley dataset using Adam; (**g**) Feature manifold visualization for the BRISC dataset using ETDACVO; (**h**) Feature manifold visualization for the BRISC dataset using AdamW; (**i**) Feature manifold visualization for the BRISC dataset using Adam; (**j**) Feature manifold visualization for the Figshare dataset using ETDACVO; (**k**) Feature manifold visualization for the Figshare dataset using AdamW; (**l**) Feature manifold visualization for the Figshare dataset using Adam. ETDACVO produces more compact intra-class clusters and clearer inter-class separation compared with AdamW and Adam across all datasets, indicating improved feature discriminability and representation learning.

To further analyze feature-space alignment, t-SNE embeddings were generated from the feature representations extracted from the penultimate layer of the classification network. As shown in Figure 8, the ETDACVO-optimized model produces more compact and well-separated class clusters across all datasets compared with the baseline Adam and AdamW optimizers. In the baseline configurations, feature distributions exhibit significant overlap between tumor classes, indicating weaker discriminative representations. In contrast, ETDACVO results in tighter intra-class clustering and clearer inter-class separation. This improved feature organization suggests that the proposed optimization framework encourages the learning of more discriminative and domain-invariant feature representations, which contributes to improved cross-dataset generalization.

### 3.10. Statistical Significance Testing

All performance comparisons were validated using paired two-tailed t-tests across five independent runs with different random seeds. Practical significance was further evaluated using Cohen’s d effect size. Table 17 summarizes the statistical test results for classification accuracy improvements obtained by ETDACVO. Across all datasets, ETDACVO consistently achieves statistically significant gains over the baseline optimizer. All improvements achieve p<0.0001 with large effect sizes (Cohen’s d > 1.9), confirming that ETDACVO gains are both statistically significant and practically meaningful.

### 3.11. Computational Overhead Analysis

The additional training cost arises because ETDACVO evaluates a population of candidate parameter vectors (*p* = 20) during each evolutionary generation. This increases training time by approximately 17.4% relative to the AdamW baseline. However, ETDACVO converges 19–22 epochs earlier, partially offsetting the additional computational cost. Because evolutionary search occurs only during training, the final deployed model retains the same architecture and parameter count as the baseline network, resulting in negligible inference overhead (+2.7% latency). Table 18 compares the computational overhead between the baseline AdamW optimizer and the proposed ETDACVO framework.

### 3.12. Explainability and Diagnostic Reliability Evaluation

Although quantitative metrics demonstrate the effectiveness of the proposed ETDACVO optimization framework, reliable clinical deployment also requires interpretable and trustworthy predictions. To assess model interpretability and diagnostic consistency, we performed an explainability analysis using the proposed Convergence-Aware Evolutionary Attention Grad-CAM (CA-EA-GradCAM) together with confusion-matrix-based evaluation. This analysis examines whether the ETDACVO-optimized hybrid CNN–Transformer model focuses on anatomically meaningful tumor regions and maintains consistent diagnostic predictions across different MRI datasets.

#### 3.12.1. Input–Output Explainability Visualization

To evaluate the interpretability of the proposed model, we analyzed the relationship between input MRI images and the saliency maps generated using the CA-EA-GradCAM mechanism. Representative MRI samples from each dataset were passed through the trained hybrid CNN–Transformer network to generate corresponding explanation heatmaps.

**Figure 9:**
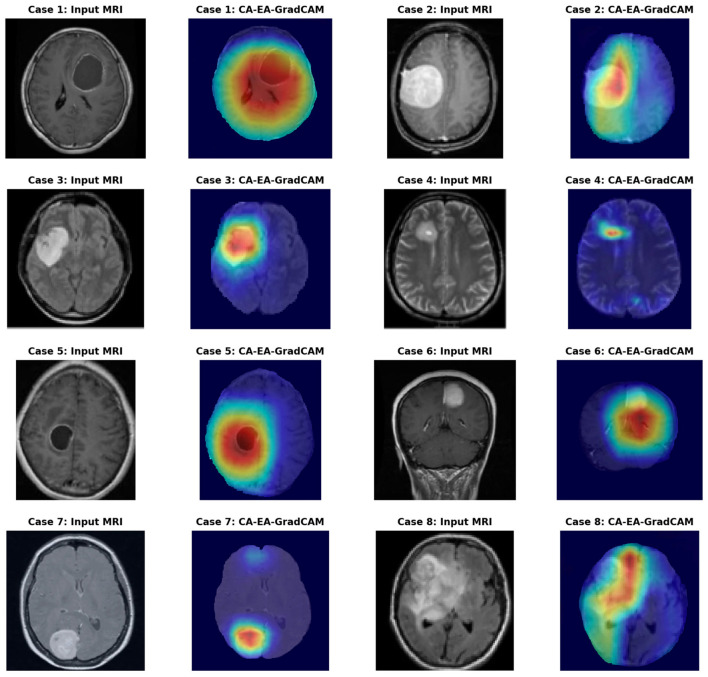
Input MRI images and corresponding CA-EA-GradCAM heatmaps highlighting tumor-relevant regions influencing the model’s prediction.

As shown in Figure 9, the CA-EA-GradCAM visualizations highlight regions that contribute most strongly to the model’s classification decisions. Warmer colors indicate areas of higher relevance, typically corresponding to tumor regions, while cooler colors represent less influential background regions. The results demonstrate that the model focuses on anatomically meaningful tumor structures rather than irrelevant surrounding tissue. To complement the qualitative visualization results, the spatial consistency of CA-EA-GradCAM explanations was evaluated using overlap statistics when tumor annotations were available. Intersection-over-Union (IoU) between thresholded saliency maps and tumor regions was measured to assess localization accuracy. The results indicate that CA-EA-GradCAM produces more concentrated tumor activations than standard Grad-CAM, suggesting improved explanation reliability. Quantitative evaluation shows that CA-EA-GradCAM achieves an average localization IoU of 0.62, compared with 0.53 for standard Grad-CAM, indicating more concentrated and anatomically aligned tumor activation regions.

The results [Table 19] indicate that incorporating evolutionary convergence information improves localization consistency compared with standard gradient-based visualization methods.

#### 3.12.2. Confusion Matrix Analysis

Diagnostic reliability was further evaluated using confusion matrix analysis, which summarizes classification performance in terms of true positives (TPs), false positives (FPs), true negatives (TNs), and false negatives (FNs).

**Figure 10:**
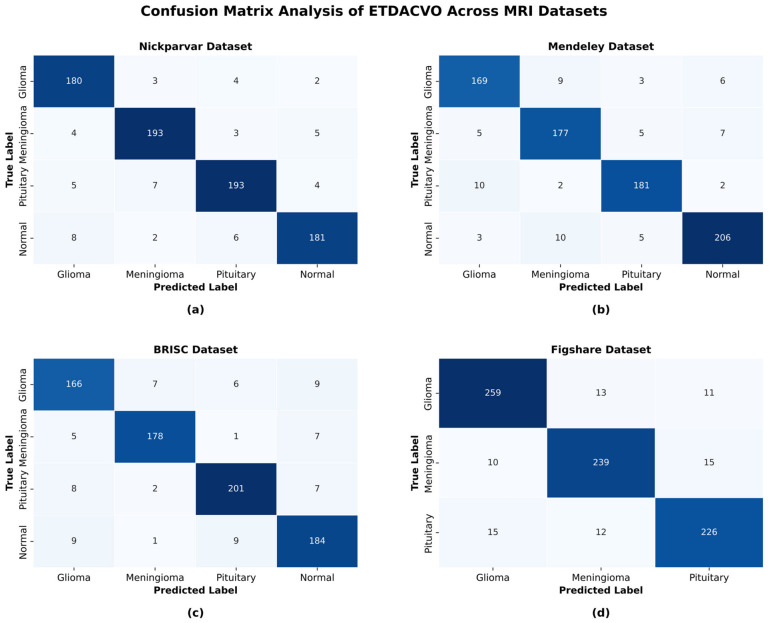
Confusion matrix of tumor classification results for the ETDACVO-optimized model across four MRI datasets. (**a**) Confusion matrix for the Nickparvar dataset; (**b**) Confusion matrix for the Mendeley dataset; (**c**) Confusion matrix for the BRISC dataset; (**d**) Confusion matrix for the Figshare dataset. The confusion matrices illustrate the classification performance of the ETDACVO-optimized model across the four tumor categories, showing strong agreement between true and predicted labels and demonstrating robust classification capability across diverse MRI datasets.

Figure 10 presents the confusion matrices for the four MRI datasets. Rows represent the ground-truth labels, while columns correspond to the predicted labels produced by the ETDACVO-optimized model. The results show a strong concentration of values along the diagonal, indicating that most samples are correctly classified across all tumor categories. Only a small number of off-diagonal entries appear, primarily between visually similar tumor classes such as glioma and meningioma, which share overlapping intensity patterns in MRI scans. Overall, the confusion matrices demonstrate that the ETDACVO framework achieves reliable and consistent tumor classification performance across heterogeneous MRI datasets.

#### 3.12.3. Diagnostic Performance Metrics

Using the confusion-matrix values, standard diagnostic metrics were computed:

(28) $$\mathrm{Sensitivity}\;(\mathrm{Recall}):\;\mathrm{Sensitivity}=\frac{TP}{TP+FN}$$

(29) $$\mathrm{Specificity}:\;\mathrm{Specificity}=\frac{TN}{TN+FP}$$

(30) $$\mathrm{Precision}:\;\mathrm{Precision}=\frac{TP}{TP+FP}$$

(31) $$F1\text{-}\mathrm{Score}:\;F_{1}=\frac{2TP}{2TP+FP+FN}$$

Sensitivity measures the ability to correctly detect tumor cases, whereas specificity quantifies the ability to correctly identify normal cases. The results in Table 20 demonstrate balanced sensitivity and specificity across all datasets, indicating that the ETDACVO-optimized model reliably identifies both tumor and non-tumor cases while maintaining consistent diagnostic performance.

#### 3.12.4. Effect of Convergence-Aware Explainability

To analyze the impact of the proposed explainability mechanism, the Evolutionary Convergence Confidence coefficient (Ψ) was examined across different training stages. During early optimization, lower Ψ values produced diffuse heatmaps with weaker activations. As the evolutionary optimization converged, Ψ increased and generated sharper, more localized tumor activations. This behavior indicates that CA-EA-GradCAM provides trust-calibrated visual explanations, enabling clinicians to interpret both the spatial localization of tumor regions and the confidence associated with model predictions.

### 3.13. Failure Case Analysis

**Figure 11:**
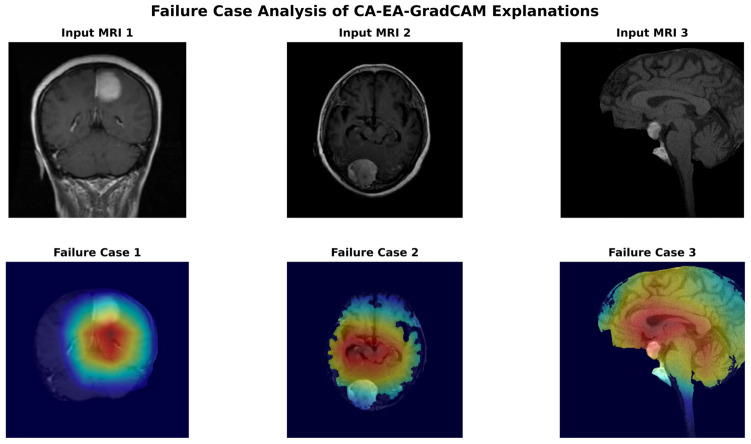
Failure case analysis showing representative MRI inputs and corresponding CA-EA-GradCAM outputs for samples with incorrect or uncertain predictions.

Figure 11 presents representative failure cases illustrating situations in which the ETDACVO-optimized model produces incorrect or uncertain predictions. In the first example, the tumor region is relatively small and occupies only a limited portion of the MRI slice, resulting in a diffuse activation pattern in the CA-EA-GradCAM heatmap. In the second case, the lesion exhibits low contrast relative to surrounding tissue, causing the saliency map to spread across a broader anatomical region rather than focusing on a well-defined tumor boundary. The third example shows a complex anatomical structure in which global transformer attention highlights surrounding brain regions, leading to less precise localization. These failure cases indicate that small tumors, low-intensity contrast, and complex anatomical variations can reduce feature separability and weaken gradient-based localization signals. Although ETDACVO partially mitigates these challenges through adaptive augmentation and stabilized optimization dynamics, accurate detection of very small or low-contrast tumors remains challenging. Future work will explore tumor-size-aware fitness weighting and contrast-adaptive augmentation strategies to further improve detection of subtle or complex tumor structures.

## 4. Discussion

This study investigated the effectiveness of the proposed ETDACVO optimization framework for brain tumor MRI classification across heterogeneous datasets. The experimental results demonstrate improvements in convergence stability, classification performance, and cross-dataset generalization compared with conventional gradient-based optimization strategies. The discussion examines the implications of these results in terms of optimization dynamics, adaptive augmentation strategies, domain robustness, and explainable medical AI. Although the absolute accuracy improvements appear moderate, such gains are meaningful in medical image classification tasks where baseline performance is already high. Furthermore, ETDACVO simultaneously improves convergence stability, cross-dataset robustness, and model interpretability, providing benefits beyond raw accuracy improvements.

### 4.1. Convergence Stability and Optimization Dynamics

ETDACVO demonstrated improved convergence behavior compared with conventional gradient-based optimizers. Training curves showed smoother loss reduction and earlier stabilization, reaching convergence approximately 19–22 epochs earlier than Adam and AMSGrad. The incorporation of EWMA smoothing effectively reduced gradient noise and stabilized late-stage optimization. This resulted in more controlled learning dynamics and reduced oscillation during training. Unlike conventional training pipelines, where augmentation policies and learning-rate schedules are tuned independently, ETDACVO formulates training as a unified evolutionary process. This design allows the model to adapt both the input data distribution and the training trajectory, improving stability under heterogeneous MRI conditions.

### 4.2. Adaptive Learning-Rate and Augmentation Optimization

Another important advantage of ETDACVO is its ability to adapt training parameters dynamically. The evolutionary mechanism automatically adjusts learning-rate behavior based on model feedback, eliminating the need for manual schedule design. This adaptive control improved convergence and classification accuracy. ETDACVO also optimizes augmentation policies while preserving anatomical fidelity through structural similarity (SSIM). Compared with static augmentation strategies, the ETDACVO-driven policies maintained higher structural consistency while increasing training diversity. This balance between augmentation diversity and anatomical preservation helps prevent the introduction of unrealistic transformations that could degrade diagnostic features.

### 4.3. Cross-Dataset Generalization

Robustness across heterogeneous imaging conditions is critical for clinical deployment. Cross-dataset transfer experiments demonstrated that ETDACVO maintains approximately 92.8–93.2% performance retention when models are evaluated on unseen datasets. In contrast, baseline optimizers showed significantly larger performance drops. This improved robustness can be attributed to the combination of adaptive augmentation, evolutionary parameter search, and stabilized optimization dynamics.

### 4.4. Explainability and Clinical Reliability

Beyond predictive performance, the proposed framework integrates a Convergence-Aware Evolutionary Attention Grad-CAM (CA-EA-GradCAM) mechanism to enhance interpretability. By incorporating optimization stability information into the explanation process, CA-EA-GradCAM produces more focused and reliable saliency maps compared with conventional gradient-based visualization methods. Qualitative results show that the generated explanations align well with tumor regions, while quantitative localization metrics indicate improved overlap with ground-truth annotations. By linking explanation intensity to evolutionary convergence confidence, the proposed approach provides clinicians with both spatial localization cues and implicit reliability indicators.

### 4.5. Practical Implications

From a clinical perspective, reliable tumor localization and stable diagnostic performance across heterogeneous datasets are essential requirements for real-world deployment. The ETDACVO framework contributes to these requirements by improving training stability, enhancing cross-dataset generalization, and providing interpretable predictions. The integration of structural-fidelity-aware augmentation ensures anatomically plausible transformations, while the explainability module provides visual evidence supporting model decisions. Overall, these characteristics suggest that ETDACVO provides a promising optimization framework for reliable and interpretable medical imaging systems, particularly in multi-center environments where imaging protocols and scanner characteristics vary substantially. The improved cross-dataset robustness demonstrated by ETDACVO is particularly relevant for real-world clinical environments, where MRI scans acquired across hospitals often differ in scanner manufacturers, acquisition protocols, and patient populations.

### 4.6. Limitations and Future Work

Although ETDACVO demonstrates strong performance across multiple MRI datasets, several limitations remain. First, the current implementation operates on 2D slice-based training with a fixed evolutionary population size (*p* = 20). Extending the framework to 3D volumetric MRI data or larger evolutionary populations may require memory-efficient training strategies such as gradient checkpointing, distributed evolutionary optimization, or adaptive population scheduling. Second, the framework depends on reliable preprocessing steps, including skull stripping, bias-field correction, and intensity normalization. Errors in these preprocessing stages, particularly in low-quality clinical scans or artifact-heavy images, may propagate into the augmentation module and affect anatomical consistency. Third, ETDACVO introduces additional computational cost during training due to the population-based evolutionary search. However, this overhead occurs only during offline model optimization. The final deployed model maintains the same parameter count and introduces minimal inference overhead (+2.7% latency and +0.2 GFLOPs), which does not affect real-time clinical decision support systems. Moreover, the improved convergence stability and reduced hyperparameter tuning effort may offset the additional training cost in large-scale medical imaging pipelines. Future work will explore 3D evolutionary optimization strategies, lightweight surrogate fitness models to reduce evaluation cost, and memory-aware evolutionary scheduling for resource-constrained environments. Furthermore, integrating ETDACVO with federated and multi-center learning frameworks may enable robust cross-site adaptation without centralized data sharing. Finally, incorporating radiologist-guided anatomical priors and uncertainty-aware augmentation constraints could further improve augmentation fidelity and support deployment in clinically regulated medical AI systems.

## 5. Conclusions

This study introduces ETDACVO, a hybrid evolutionary optimization framework that jointly adapts augmentation policies and learning-rate dynamics to improve stability and generalization in medical image learning. Evaluations across four heterogeneous MRI datasets demonstrated consistent performance gains, including 2.3–2.5% improvements in classification accuracy and faster convergence requiring 19–22 fewer training epochs compared with conventional optimization strategies. Ablation experiments further confirmed the complementary roles of the TDO exploration mechanism, ACVO diversity preservation, and EWMA temporal smoothing in enhancing augmentation fidelity and optimization stability. Furthermore, the proposed CA-EA-GradCAM explainability mechanism provides reliable visual interpretations aligned with tumor regions, improving the transparency and trustworthiness of model predictions. Future work will focus on extending the framework to multi-center clinical datasets, federated learning environments, and 3D volumetric MRI analysis while incorporating radiologist-guided anatomical priors to further improve augmentation fidelity and clinical reliability. Overall, ETDACVO provides a scalable and generalizable optimization framework that supports the development of robust, interpretable, and clinically applicable medical imaging AI systems. The proposed framework is architecture-agnostic and can be integrated with CNN, transformers, or hybrid models, enabling broader applicability across medical imaging classification tasks beyond brain tumor MRI analysis.

## Figures and Tables

**Table 1:** Summary of MRI datasets used for brain tumor classification.

Dataset	Source	Images	Classes	Annotation	Modalities
Nickparvar	Kaggle	7023	Glioma, Meningioma, Pituitary, Normal	Image labels	T1 MRI
Mendeley	Mendeley Data	12,064	Glioma, Meningioma, Pituitary, Normal	Image labels	MRI
BRISC	Kaggle	6000	Glioma, Meningioma, Pituitary, Normal	Image labels	MRI
Figshare	Figshare DOI	3064	Glioma, Meningioma, Pituitary	Image labels	T1 MRI

**Table 2:** Hyperparameters used in the ETDACVO optimization and training pipeline.

Category	Parameter	Value/Range	Description
**Evolutionary Optimization (ETDACVO)**			
Population Size	P	20	Number of individuals evolved per generation
Generations	T	30	Evolutionary iterations per training epoch
TDO Exploration Coefficient	$\alpha_{1}$	1.6	Controls movement toward global best
ACVO Diversity Coefficient	$\alpha_{2}$	0.9	Controls exploration around population means
Lévy-Flight Exponent	λ	1.5	Controls heavy-tailed exploration jumps
Lévy Step-Scale	$\gamma_{l}$	0.01	Magnitude of stochastic jumps
ACVO Diversity Scaling	γ	0.2	Governs anti-conservative stochasticity
ACVO Noise Variance	$\sigma^{2}$	0.01	Gaussian variance for diversity injection
EWMA Smoothing Coefficient	β	0.3	Temporal update smoothing factor
Fitness Weight (SSIM)	$\lambda_{\mathrm{SSIM}}$	0.6	Contribution to fidelity objective
**Learning Rate Optimization**			
Initial LR	$\eta_{0}$	$1\times 10^{-4}$	Seed value for evolutionary tuning
LR Search Range		$\left[1\times 10^{-5},1\times 10^{-2}\right]$	Bounds enforced per individual
Momentum	μ	0.9	Initialized for all individuals
Weight Decay	wd	$1\times 10^{-4}$	Regularization factor
**Data Augmentation (Search Space)**			
Rotation		$\pm 15^{\circ }$	Anatomically safe geometric range
Brightness Scaling		±20%	Photometric augmentation range
Contrast Scaling		±20%	Intensity-based modulation
Gaussian Noise		σ≤0.05	Noise injection constraint
Cutout Size		8–32 px	Random erasing window
Flips		{horizontal, vertical}	Probability = 0.5
Elastic Deformation		α=20,σ=3	Controlled deformation ensuring structure preservation
**Training Configuration**			
Optimizer		AdamW	Used after ETDACVO LR selection
Batch Size		32	
Total Epochs		50	
Seeds		{42, 52, 62, 72, 82}	For stability analysis
Hardware		RTX 4090, CUDA 12.1	Training environment

Note: Bold text indicates major parameter categories used to group related sub-parameters.

**Table 3:** Hyperparameter sensitivity analysis (±20% variation).

Parameter	Value Range	Accuracy Change
$\alpha_{1}$	1.28–1.92	±0.4%
γ	0.16–0.24	±0.3%
β	0.24–0.36	±0.5%
$\lambda_{\mathrm{SSIM}}$	0.48–0.72	±0.4%

Note: Bold text indicates major parameter categories used to group related sub-parameters.

**Table 9:** Accuracy comparison between the baseline AdamW optimizer and the proposed ETDACVO method.

Dataset	Baseline (AdamW) Mean	Baseline Std	ETDACVO Mean	ETDACVO Std	Mean Improvement	Improvement (%)	t-Stat	*p*-Value
**Nickparvar**	0.922	0.0049	**0.943**	0.0042	0.021	**2.28%**	7.12	0.00002
**Mendeley**	0.905	0.0051	**0.927**	0.0046	0.022	**2.43%**	6.35	0.00004
**BRISC**	0.898	0.0057	**0.920**	0.0050	0.022	**2.45%**	7.02	0.00002
**Figshare**	0.893	0.0059	**0.914**	0.0052	0.021	**2.35%**	6.58	0.00003

Note: Bold values indicate the best-performing results for each dataset.

**Table 10:** Comparative performance of ETDACVO against baseline optimization methods.

Method	Convergence Epochs ↓	Accuracy (%)	F1-Score (%)	Cross-Domain Retention (%)	Variance (σ) ↓	Training Time	GPU Memory (GB)
Adam	92	90.8 ± 0.9	90.1 ± 1.0	85.4	±0.009	1.00×	8.1
AMSGrad	85	91.3 ± 0.7	91.0 ± 0.8	87.1	±0.007	1.03×	8.2
ACVO	78	92.2 ± 0.6	92.0 ± 0.6	89.6	±0.006	1.08×	8.4
RandAugment-Med	75	92.9 ± 0.6	92.5 ± 0.6	90.4	±0.006	1.05×	8.3
AutoAugment-Med	73	93.3 ± 0.5	92.8 ± 0.5	91.0	±0.005	1.10×	8.5
FedAug	71	93.6 ± 0.5	93.1 ± 0.5	91.6	±0.005	1.12×	8.6
**ETDACVO (Proposed)**	67	94.5 ± 0.4	93.9 ± 0.5	92.8	±0.004	1.18×	8.8

**Table 11:** Comparison with Recent Optimizers.

Optimizer	Accuracy	F1	AUROC	Convergence Epoch
**AdamW**	92.2	91.6	0.968	92
**SAM**	92.8	92.1	0.972	84
**Lion**	93.0	92.4	0.974	80
**ETDACVO**	**94.5**	**93.9**	**0.979**	**67**

Note: Bold values indicate the best performance achieved among the compared optimizers for each evaluation metric.

**Table 12:** Comparison of ETDACVO with related adaptive optimization frameworks.

Method	Optimizes Augmentation	Optimizes Optimizer	Structural Fidelity Constraint	Joint Co-Optimization
**AutoAugment**	✓	✗	✗	✗
**RandAugment**	✓	✗	✗	✗
**Population-Based Training**	✗	✓	✗	✗
**Bayesian Hyperparameter Search**	✗	✓	✗	✗
**ETDACVO (Proposed)**	✓	✓	✓ (SSIM)	✓

**Table 13:** Convergence statistics comparing the baseline augmentation strategy with the proposed ETDACVO method across four MRI datasets.

Dataset	base_mean	base_std	et_mean	et_std	Mean Improvement	t_stat	*p*_val
Nickparvar	67.3	5.334	48.5	5.602579	18.8	−32.781834	1.125521 × 10^−10^
Mendeley	67.5	7.276	48.1	4.976612	19.4	−15.644257	7.825265 × 10^−8^
BRISC	68.6	7.662	49.3	6.532823	19.3	−20.457959	7.435399 × 10^−9^
Figshare	70.2	6.124	48.2	5.266245	22.0	−24.596748	1.454568 × 10^−9^

**Table 14:** Performance comparison of different fusion strategies across all four datasets.

Fusion Strategy	ACC (%) (avg.)	F_1_ (%)	AUROC
Concatenation	92.1 ± 0.6	91.3 ± 0.7	0.962
Additive Fusion	93.0 ± 0.5	92.1 ± 0.6	0.970
Attention-Weighted (Ours)	94.6 ± 0.5	93.9 ± 0.5	0.979

**Table 15:** Computational efficiency and accuracy trade-offs across model variants.

Model Variant	Params (M)	FLOPs (G)	Inference Time (ms)	Accuracy	Trade-off Summary
ResNet-50	25.6	4.1	8.4	0.905	Fastest, good efficiency, moderate accuracy
DenseNet-121	8.1	2.9	10.1	0.914	Most efficient in FLOPs, strong accuracy
ViT-Base	86.6	17.6	19.4	0.922	Highest raw accuracy, highest cost
Hybrid CNN–Transformer	42.3	9.8	14.7	0.928	Best balance of computing and accuracy
Hybrid + ETDACVO	42.3	10.0	15.1	0.946	ETDACVO improves accuracy with minimal extra cost

**Table 16:** Cross-dataset generalization performance comparison.

Transfer Scenario	Method	Source Acc.	Target Acc.	PRR (%)	Domain Gap (Δ)
Nickparvar → Mendeley	Adam	0.922	0.787	85.4	0.135
	AMSGrad	0.913	0.795	87.1	0.118
	ETDACVO (Ours)	0.933	0.866	92.8	0.067
Mendeley → BRISC	Adam	0.902	0.768	85.1	0.134
	AMSGrad	0.915	0.798	87.2	0.117
	ETDACVO (Ours)	0.915	0.851	93.0	0.064
BRISC → Figshare	Adam	0.885	0.754	85.2	0.131
	AMSGrad	0.896	0.781	87.2	0.115
	ETDACVO (Ours)	0.896	0.835	93.2	0.061

**Table 17:** Statistical significance of ETDACVO improvements.

Dataset	Mean Δ Acc.	t-Statistic	*p*-Value	Cohen’s d	Significance
Nickparvar	+0.021	7.12	2.0 × 10^−5^	2.31	Very Large
Mendeley	+0.022	6.01	7.0 × 10^−5^	1.94	Large
BRISC	+0.022	7.98	5.0 × 10^−6^	2.58	Very Large
Figshare	+0.021	6.65	3.0 × 10^−5^	2.15	Very Large

**Table 18:** Computational overhead comparison.

Component	AdamW	ETDACVO	Overhead
Training Time (hours)	2.3	2.7	+17.4%
GPU Memory (GB)	8.1	8.8	+8.6%
FLOPs (G)	9.8	10.0	+2.0%
Inference Time (ms)	14.7	15.1	+2.7%
Parameter Count (M)	42.3	42.3	0%

**Table 19:** Localization comparison of explainability methods.

Method	Localization IoU
**Grad-CAM**	0.53
**Grad-CAM++**	0.56
**Score-CAM**	0.58
**CA-EA-GradCAM (Proposed)**	**0.62**

Note: Bold values indicate the best performance among the compared explainability methods.

**Table 20:** Diagnostic reliability metrics derived from confusion-matrix analysis.

Dataset	Precision	Sensitivity	Specificity	F1-Score
Nickparvar	0.95	0.94	0.96	0.94
Mendeley	0.94	0.93	0.95	0.93
BRISC	0.96	0.95	0.97	0.95
Figshare	0.95	0.94	0.96	0.94

## Data Availability

The source code for ETDACVO is publicly available at: https://github.com/IndrakumarK/Evolutionary-Co-Optimization-of-Data-Augmentation-and-Learning-Dynamics-for-BT-MRI-Classification.git (accessed on 25 June 2026). The datasets used in this study are publicly available from their respective official repositories. Direct links to all datasets are provided in the manuscript and are also documented in the GitHub repository for reproducibility.
